# Smartphone-Based Remote Monitoring for Cardiac Implantable Electronic Devices: Transmission Performance and Early Alert Responsiveness

**DOI:** 10.1016/j.jacasi.2026.04.028

**Published:** 2026-07-07

**Authors:** Satoshi Yanagisawa, Yasuya Inden, Michiko Hibino, Yuki Sato, Yuji Narita, Hiroyuki Miyazawa, Kiichi Miyamae, Taishi Fukushima, Takehiro Hiramatsu, Kentaro Adachi, Tsubasa Teraoka, Ryusuke Ota, Yuki Tanaka, Masafumi Shimojo, Yukiomi Tsuji, Takahiro Okumura, Toyoaki Murohara

**Affiliations:** a Department of Advanced Cardiovascular Therapeutics, Nagoya University Graduate School of Medicine, Nagoya, Japan; b Department of Cardiology, Nagoya University Graduate School of Medicine, Nagoya, Japan; c Department of Clinical Engineering, Nagoya University Hospital, Nagoya, Japan; d Department of Cardiac Surgery, Nagoya University Graduate School of Medicine, Nagoya, Japan

**Keywords:** cardiac implantable electronic devices, mobile app, MyCareLink Heart app, remote monitoring, transmission, ventricular tachycardia

Mobile app-based remote monitoring (RM) for cardiac implantable electronic devices (CIEDs) has emerged as a next-generation alternative to conventional bedside console systems. By leveraging personal smartphones, app-based RM enables continuous connectivity and potentially more timely detection of significant events, particularly when patients are away from home. Although adoption has expanded in North America and Europe,^1^, ^2^, ^3^, ^4^ implementation across Asia remains extremely limited. By 2025, the estimated penetration rate in Japan is expected to be remarkably low. We therefore evaluated the clinical implementation of the Medtronic MyCareLink Heart app in a tertiary CIED center in Japan, focusing on transmission performance, alert responsiveness, and clinical usability.

## Methods

The study population comprised patients who began using the MyCareLink Heart app at Nagoya University Hospital, Japan, between January 2019 and October 2025. Eligible patients had previously implanted Medtronic CIEDs and access to a compatible smartphone (currently limited to iPhone devices in Japan). Patients transitioning from a traditional bedside monitoring system were included. A matched control group using the conventional home communicator system (MyCareLink Relay) was constructed from our institutional CIED database comprising 4,484 patients. Matching criteria included age (±5 years), sex, RM duration, and device type in a 1:1 ratio. During installation, a certified clinical engineer evaluated patient independence across 10 predefined essential tasks required to activate and operationalize the app (eg, password setup, device identification, download process). Each successfully completed task was assigned 1 point (range 0-10).

Devices were programmed for automatic monthly scheduled transmissions.^5^ In addition, the patients were asked to visit the outpatient clinic for face-to-face interactions every 6 to 12 months. We generally did not contact patients when scheduled transmission was unavailable. Instead, we asked about RM inaccessibility during visits and checked their connection status. The study protocol was approved by the institutional ethics committee at Nagoya University Hospital (approval no. 2015-0192) and written informed consent was obtained in accordance with the Declaration of Helsinki. Data are presented as n (%), mean ± SD, or median (IQR). Differences were assessed using Student’s *t*-test or the Mann-Whitney *U* test, and categorical variables using the chi-square or Fisher’s exact test. Paired comparisons used the Wilcoxon signed-rank test, and comparisons among >2 groups used 1-way analysis of variance. A *P* value <0.05 was considered statistically significant. Analyses were performed using SPSS version 28.0 (SPSS Inc).

## Results

**Figure 1:**
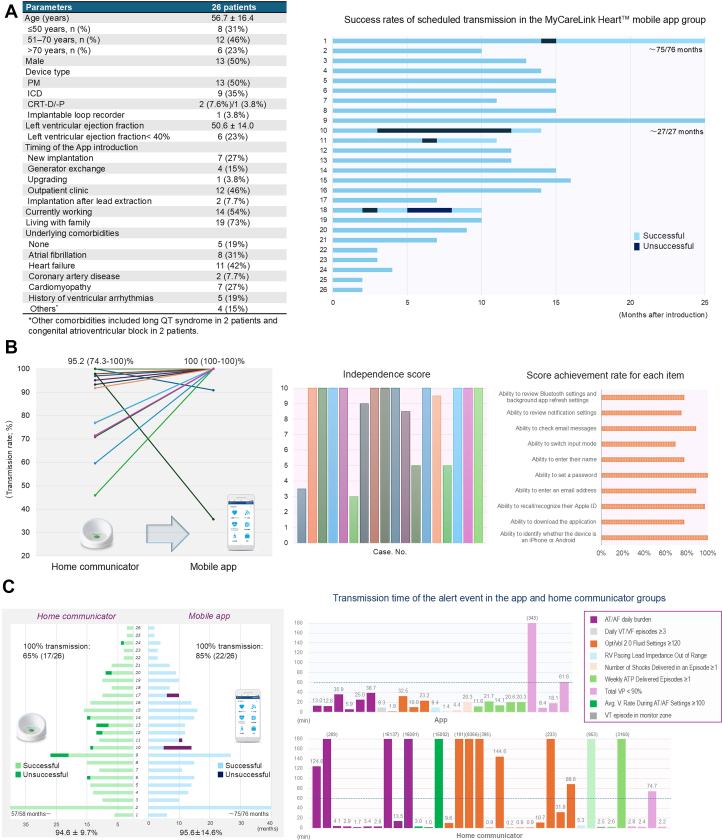
Baseline Characteristics, Successful Transmission Rates, Independence Score, and Alert Events (A) Baseline characteristics and success rates of scheduled transmissions in the MyCareLink Heart mobile app. Two patients (nos. 10 and 18) had a low success rate of transmissions. The device of 1 patient was presumed to have been security-locked because of an iOS update or communication disruptions occurred. Another patient had a continuous connection error during monitoring; however, communication was restored after enabling Bluetooth, relaunching the application, and restarting the iPhone. (B) Transmission rate after transitioning from home communicator monitoring to the app (left); and independence score and achievement of each item (right). (C) Success rates of scheduled transmissions and transmission time of the alert event between the app and home communicator groups. Permission was obtained from Medtronic Japan to use the mobile app and home communicator images. AF = atrial fibrillation; ATP = antitachycardia pacing; CRTD = cardiac resynchronization therapy defibrillator; ICD = implantable cardioverter-defibrillator; PM = pacemaker; VT = ventricular tachycardia.

Twenty-six patients were enrolled (mean age 56.7 ± 16.4 years), of whom 23% (6 of 26) were older than 70 years (Figure 1A). Most patients (50%) had pacemaker devices. During a median follow-up of 11.0 (6.8-14.3) months, overall scheduled transmission success was 95.6% ± 14.6%, with 22 patients achieving 100% completion (Figure 1A). Seventeen patients transitioned from bedside console monitoring to app-based RM. Scheduled transmission rates improved numerically from 95.2% (74.3-100) to 100% (100-100) (*P* = 0.064) (Figure 1B). When console performance was restricted to a follow-up duration equivalent to that of the app to mitigate duration bias, transmission rates remained comparable (93.1% [83.3-100] vs 100% [100-100]; *P* = 0.109).

Independence scores were available in 18 patients. The mean total score was 8.52 ± 2.49, and 11 patients completed all installation steps independently (Figure 1B). In contrast, 2 patients had low scores of 3.0 and 3.5. Tasks most frequently completed without assistance included password configuration and device identification, whereas app download and input mode switching required more support. Patients were stratified into high (score 10; n = 11), median (5.1–9.9; n = 3), and low (≤5; n = 4) independence groups. Although the mean age was significantly higher in the low score group than in the other groups (75.0 ± 7.4, 69.3 ± 5.0, and 49.5 ± 13.5 years in the low, median, and high score groups, respectively, *P* = 0.003), the transmission rates did not significantly differ among the groups (99.7% ± 0.7%, 100%, and 89.6% ± 21.6%, *P* = 0.509).

In matched comparisons (n = 26 per group), scheduled transmission success was similar between app and console groups (95.6% ± 14.6% vs 94.6% ± 9.7%; *P* = 0.765) (Figure 1C). A total of 24 and 35 alert events occurred in the app and control groups, respectively. Median alert transmission times were 16.1 (9.3-24.6) minutes and 9.6 (2.5-209.4) minutes, respectively (*P* = 0.740). However, the proportion of alerts successfully transmitted within 60 minutes was significantly higher in the app group (92% [22 of 24] vs 60% [21 of 35]; *P* = 0.007) (Figure 1C). Furthermore, focusing on ventricular arrhythmia alert events, the success rate remained higher in the app group than in the control group (100% [9 of 9] vs 33% [1 of 3], *P* = 0.045).

## Discussion

App-based RM matches the needs and trends of the current information society, with the expanded use of personal smartphones and tablets. According to the 2024 Communications Usage Trend Survey in Japan,^6^ smartphone use continues to rise, reaching an individual prevalence of 80.5%. However, this trend declined with age, to 67.5% in those aged 70 to 79 years and 30.7% in those aged ≥80 years. As many CIED recipients are older, evaluating utility in this population is essential. In addition, older individuals may be less familiar with smartphones, potentially limiting app installation. Our results address this issue by demonstrating that a low independence score during the app download process was associated with older patients, and that the substantial need for staff assistance may contribute to the low penetration of app use in Japan. However, once the older patient successfully installed the app, the transmission rate was consistently high thereafter, suggesting the possibility of a long-term connection (this population probably did not touch their smartphone device frequently; therefore, the “communicated” status could be maintained).

Our study highlighted another merit of app-based RM by reporting a significantly higher rate of alert transmission within 60 minutes than with traditional RM. This finding shows that the app-based RM can successfully transmit real-time alert events, boosting patient confidence since the RM remains connected to the implanted device. Early detection of ventricular arrhythmias is crucial for prompt treatment and diagnosis to prevent deterioration of heart failure and electrical storm, leading to a better prognosis.^7^^,^^8^ Although this app is currently available only to patients using iPhones and not to those using Android devices in Japan, which may limit the generalizability of our findings, our results may provide pioneering evidence that contributes meaningfully to the advancement and dissemination of app-based RM in Asia.

### Data Availability

The de-identified participant data will not be shared.

## Funding Support and Author Disclosures

Drs Yanagisawa and Okumura are affiliated with a department sponsored by Medtronic Japan. All other authors have reported that they have no relationships relevant to the contents of this paper to disclose.
